# *OIP5* Is a Novel Prognostic Biomarker in Clear Cell Renal Cell Cancer Correlating With Immune Infiltrates

**DOI:** 10.3389/fimmu.2022.805552

**Published:** 2022-02-15

**Authors:** Mancheng Gong, Yongxiang Li, Erlin Song, Miaoyuan Li, Shaopeng Qiu, Wenjing Dong, Runqiang Yuan

**Affiliations:** ^1^ Department of Urology, The People’s Hospital of Zhongshan, Zhongshan, China; ^2^ Department of Urology, Weifang People’s Hospital, Weifang, China; ^3^ Department of Urology, The First Affiliated Hospital of Harbin Medical University, Harbin, China; ^4^ Department of Urology, The First Affiliated Hospital of Sun Yat-sen University, Guangzhou, China; ^5^ Department of Oncology, The People’s Hospital of Zhongshan, Zhongshan, China

**Keywords:** *OIP5*, ccRCC, immune cells, prognosis, survival

## Abstract

Opa interacting protein 5 (*OIP5*), overexpressed in some types of human cancers, has been reported to be associated with the carcinogenesis of human cancer. However, its contribution to cancer immunity remains unknown. Furthermore, the relationship between *OIP5* and cancer immunity remains uncertain. In our research, we explored the different expression of *OIP5* between 539 ccRCC and 72 normal renal tissues base on TCGA data set. We analyzed the associations between OIP5 expression with ccRCC progression and survival. Next, we compared immune cell profiles in cancer tissues and normal tissues in the Cancer Genome Atlas (TCGA) ccRCC cohort. We found that the level of immune cell infiltration was correlated with the copy number of *OIP5* gene in ccRCC. The effect of *OIP5* on immune activity was verified by Gene Set Enrichment Analysis of RNA-seq data from 32 ccRCC cell lines in the public database. Moreover, a pathway enrichment analysis of 49 *OIP5*-associated immunomodulators demonstrated the involvement of the T cell receptor signaling pathway, the JAK-STAT signaling pathway, the NF-kappa B signaling pathway and the primary immunodeficiency pathway. In addition, using *OIP5*-associated immunomodulators, we constructed multiple-gene risk prediction signatures using the Cox regression model. Our results provided insights into the role of *OIP5* in tumor immunity and revealed that *OIP5* may be a potential immunotherapeutic target for ccRCC. Designated immune signature is a promising prognostic biomarker in ccRCC.

## Introduction

Renal cell carcinoma accounts for 4.2% of all new cancer cases and is one of the most common urinary tumors in the world. In 2019, it was estimated that there were 73,750 new diagnoses of renal cancer and 14,830 will die of this disease in the United States (1). Clear cell renal cell carcinoma (ccRCC) accounts for 85% of all renal cancers and is the most common type of renal cancer (2). In most cases, radiotherapy and chemotherapy are less effective in the treatment of ccRCC. Radical nephrectomy is the first choice for early localized renal cancer, although it still does not solve the problem of distant metastasis or tumor recurrence in more than 20% of patients after surgery (3). Moreover, the prognosis of ccRCC with metastasis is relatively poor, with a median survival time of only about 10 months (4).

As a new treatment for ccRCC, immunotherapy is complementary to surgery, chemotherapy, and targeted therapies. In the past few decades, the treatment of renal cancer has undergone a transition from a nonspecific immune approach to vascular endothelial growth factor (VEGF) targeting therapy, and now immunotherapy is one of the newest treatment methods for renal cancer (5). In recent years, immunotherapy has made significant progress in the treatment of metastatic renal cell carcinoma. Compared to non-specific immunotherapies for IL-2 or IL-6, immune checkpoint blockade (ICB) therapy has performed very well in the treatment of advanced RCC since it was approved as second-line therapy in 2015. A clinical trial demonstrated that the PD-L1 inhibitor avelumab and the PD-1 inhibitor pembrolizumab plus axinib with that of sunitinib had superior advantages contrasted to sunitinib in RCC (6). However, it is estimated that only about 20% of patients will benefit from immunotherapy (7). Therefore, it is necessary to use predictive biomarkers to initially assess a patient’s potential response to ICB treatment.

*OIP5* was first identified as an Opa (Neisseria gonorrhoeae opacity-associated) interacting protein by yeast two-hybrid analysis (8). *OIP5* encodes a 25 kDa protein with a coiled domain, also named MIS18Beta and Lint-25. Notably, abnormal regulation of *OIP5* is commonly seen in a variety of tumors, such as glioblastoma (9), bladder cancer (10), breast cancer (11) and gastric cancer (12). Currently, it has been proved that the up-regulation of *OIP5* plays a crucial role in development of tumors, which has been confirmed *in vivo* and *in vitro*. We have reported that *OIP5* was overexpression in ccRCC in 2013 (13). However, up to now, the specific mechanism of *OIP5* in ccRCC has not been studied, and the relationship between *OIP5* and immunity has not been explored. In this study, using the TCGA database, we further proved that *OIP5* was highly expressed in ccRCC, and that *OIP5* was significantly correlated with the grade of ccRCC and the prognosis of patients. Then, we systematically assessed the status of lymphocytes and elucidate the relationship between *OIP5* and ccRCC immunity, as well as the signaling pathways that regulate *OIP5*-mediated immune response. Finally, we used *OIP5*-associated immunomodulators to generate prognostic immune signatures.

## Materials and Methods

### Acquirement of ccRCC Expression Profiles From TCGA Datasets and Bioinformatics Analysis

We obtained ccRCC dataset from the TCGA project (https://cancergenome.nih.gov/). The ccRCC dataset contained 539 cancerous and 72 normal tissues, accompanied by clinical information. RNA expression data and clinic data were further processed using R software.

### Detection of Tumor Infiltrating Immune Cells in TCGA Renal Clear Cell Carcinoma

CIBERSORT (http://cibersort.stanford.edu/) is a kind of analysis tool, which provides an estimation of the abundances of immune cells in mixed cell populations, using gene expression data (14). We identified and quantified 22 immune cells in tissues, including seven T cell types, naïve and memory B cells, plasma cells, NK cells, and myeloid subsets, by Estimating Relative Subsets Of RNA Transcripts (CIBERSORT) (14, 15). LM22 is adopted, which is a leukocyte gene signature matrix. This document contains 547 genes that distinguish 22 human hematopoietic cell phenotypes (14). Using CIBERSORT L22 as a reference, we investigated the mRNA expression matrix with the CIBERSORT R script obtained from the CIBERSORT website。

### Relationship Between *OIP5* and Tumor Immune Cell Infiltration

Tumor Immune Estimation Resource furnished a detailed data of tumor immune cells of pan-cancer (cistrome.dfci.harvard.edu/TIMER/) (16). This website provides several modules, including Survival, Gene, Mutations, Diff Exp, SCNA, Estimation and Correlation. We investigated the relationship between immune cell infiltration and *OIP5* copy numbers.

### Gene Set Enrichment Analysis

Gene Set Enrichment Analysis (GSEA) is a method to determine whether predefined gene sets are differentially expressed in different phenotypes. The Cancer Cell Line Encyclopedia (CCLE) dataset is sponsored and maintained by the Broad Institute, the Novartis Biomedical Research Institute and the Genomics Institute of the Novartis Research Foundation. This database contains most of the common cancer types, including kidney cancer, breast cancer, liver cancer, stomach cancer and lung cancer. The RNAseq data of 32 ccRCC cell lines was downloaded from the CCLE website. The expression level of *OIP5* in various renal clear cell carcinomas was extracted for further analysis. Taking the average expression of *OIP5* gene as a cutoff value, all ccRCC cell lines were divided into *OIP5* ^high^ group and *OIP5* ^low^ group. In this study, GSEA was used to explore the significant signal pathways associated with the *OIP5* gene.

### Immunomodulators

**Figure 1:**
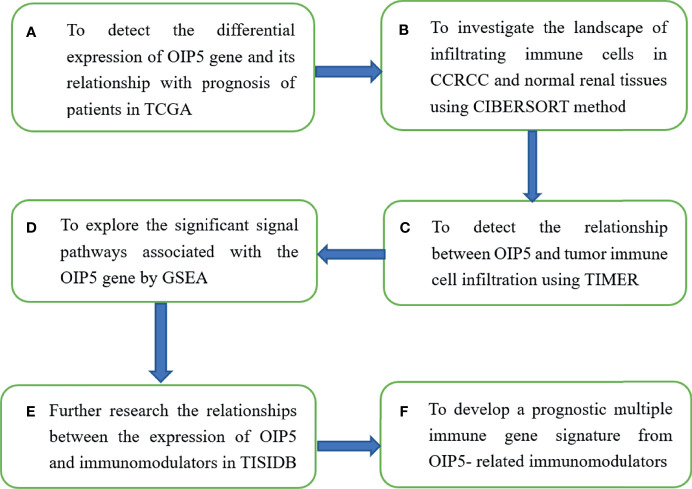
The flowchart of the research scheme of the article.

TISIDB database (http://cis.hku.hk/TISIDB) was adopted to further research the relationships between the expression of *OIP5* and immunomodulators. The TISIDB database covers the interaction between tumors and the immune system, including high-throughput screening techniques, 988 reported immune-related anti-tumor genes, molecular maps and paracancerous data sets, as well as a variety of immune data resources obtained from seven public databases (17). We recruited immunoinhibitors and immunostimulators which were significantly associated with *OIP5* regarding gene expression (Spearman correlation test, P < 0.05). Then, we analyzed the *OIP5*-associated immunomodulators using the cBioPortal for Cancer Genomics (www.cbioportal.org). Using two WEB-based GEne SeT AnaLysis Toolkit (http://www.webgestalt.org/) and web-based tools (https://string-db.org/) (18, 19), the resulting protein network was subjected to Kyoto Encyclopedia of Genes and Genomes (KEGG) pathway enrichment analysis and GO annotation (**Figure 1**).

### Survival Analysis

Next, we developed a prognostic multiple immune gene signature from OIP5-related immunomodulators. Akaike Information Criterion was used to select stepwise variables in Cox model (20). After identifying the immune genes, the prognostic index, namely risk score, was obtained: risk score =β1x1 + β2x2 +… + βixi. In this algorithm, xi is the expression level of each gene, and βi is the risk coefficient of each gene in the Cox model. Then, Kaplan–Meier survival curve was performed to analysis the relationship between risk score and patient survival (**Figure 1**).

### Statistical Analysis

All statistical analysis were conducted by R software 3.6.2. (R Foundation for Statistical Computing, Vienna, Austria) and IBM SPSS Statistics 26.0 (IBM, Inc., Armonk, NY, USA). Comparisons for continuous variables among groups ≥3 were performed by One-way ANOVA. *P* < 0.05 was considered to be statistically significant.

## Result

### Increased Expression of *OIP5* in ccRCC Tissues

**Figure 2:**
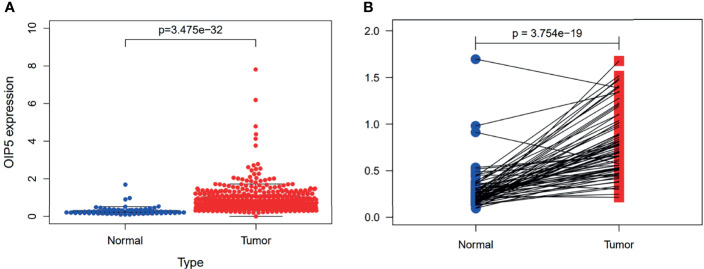
Expression of *OIP5* in 539 ccRCC and 72 normal renal tissues. **(A)** The different expression of *OIP5* in 539 ccRCC and 72 normal renal tissues. **(B)** The differential expression of *OIP5* in 72 pairs of ccRCC tissues and adjacent non-tumor tissues.

The gene expressions data of 539 ccRCC and 72 normal renal tissues were downloaded from TCGA. Our results revealed the *OIP5* gene was highly expressed in ccRCC compared to normal renal tissues (*P* < 0.001) (**Figure 2A**). Furthermore, the paired test also confirmed that *OIP5* was highly expressed in ccRCC (N=72, *P* < 0.001) (**Figure 2B**).

### Relationship Between *OIP5* Expression and the Clinicopathological Features of ccRCC

**Figure 3:**
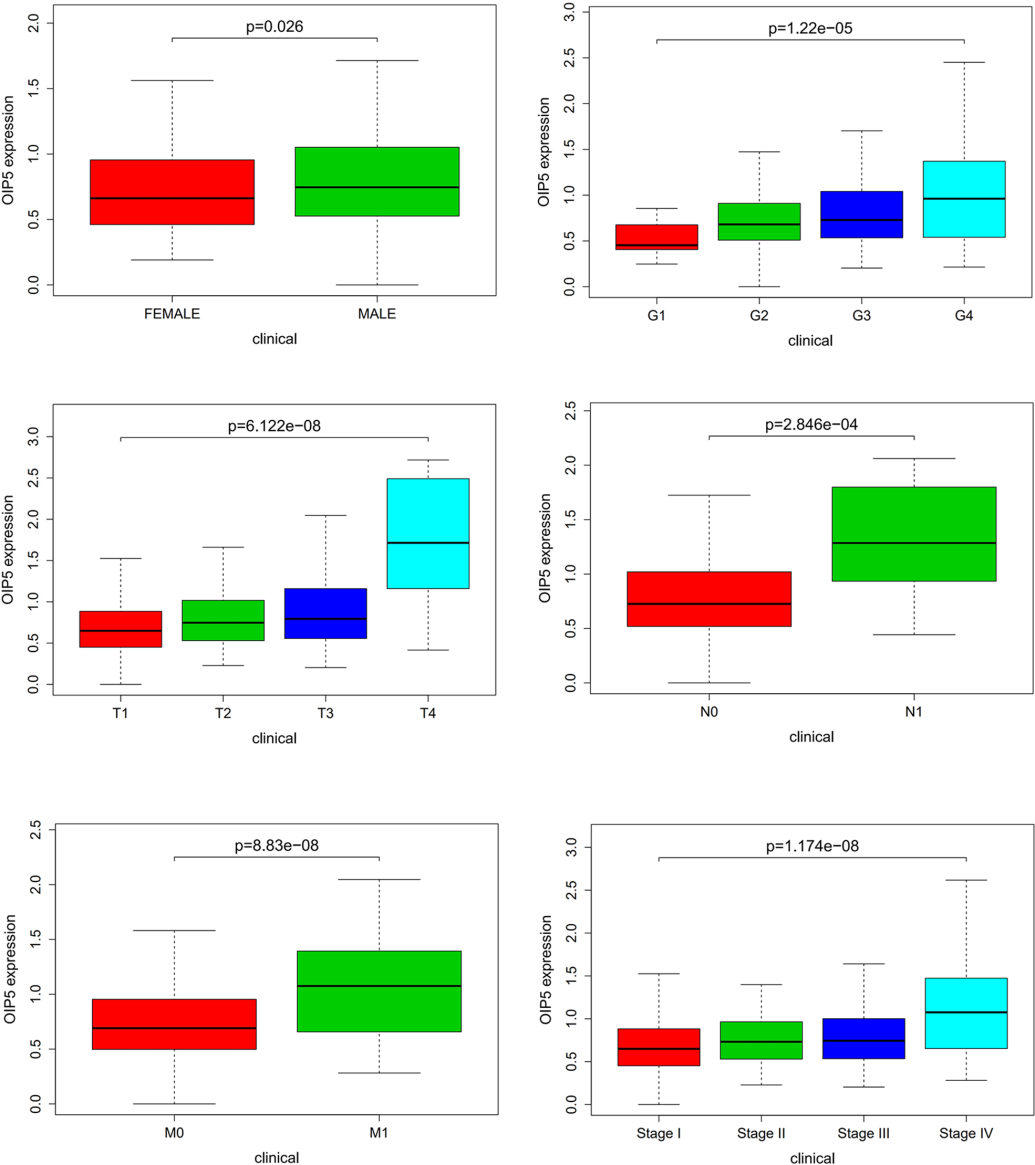
Relationship between *OIP5* expression and the clinicopathological features of ccRCC.

A total of 539 ccRCC tissues with *OIP5* expression data across all patient characteristics were analyzed from TCGA. As shown in **Figure 3**, high expression of *OIP5* gene correlated significantly with the patient’s age (*P* =0.026), the tumor histological grade (*P <*0.001), T classification (*P <*0.001), N classification (*P <*0.001), M classification (*P <*0.001) and clinical stage (*P <*0.001).

### Impact of *OIP5* Overexpression on Overall Survival in ccRCC

**Figure 4:**
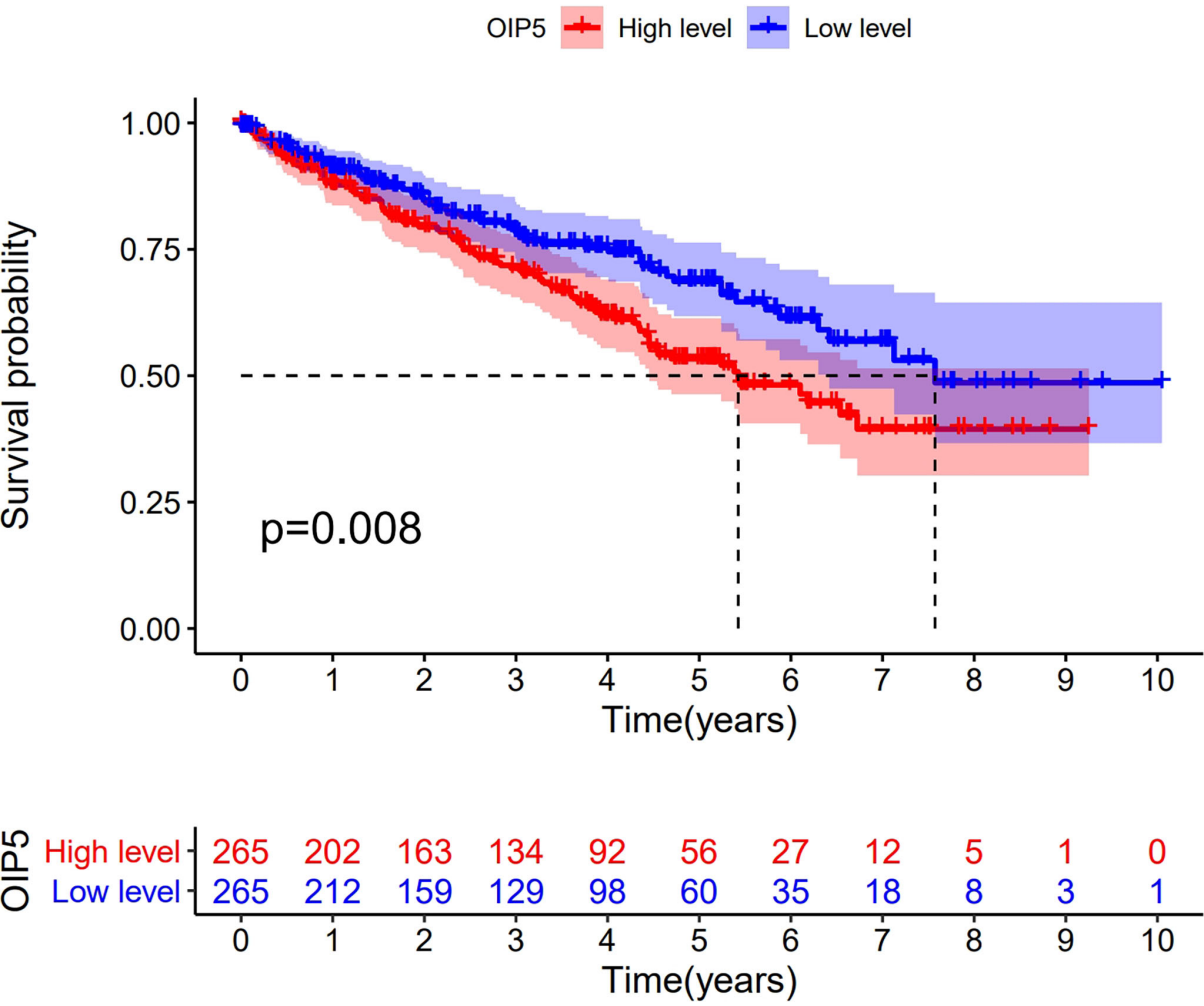
Impact of *OIP5* overexpression on overall survival in ccRCC.

Kaplan-Meier survival analysis demonstrated that ccRCC with *OIP5*-high expression had a worse prognosis than that with *OIP5*-low expression (**Figure 4**, *P* = 0.008). To explore the impact of *OIP5* overexpression and other clinicopathological parameters in ccRCC patients, we performed univariate and multivariate analyses. The univariate analysis revealed that *OIP5* overexpression (*P* < 0.001), age (*P* < 0.001), grade (*P* < 0.001), stage (*P* < 0.001), T classification (*P* < 0.001) and M classification (*P* < 0.001) were all positively associated with a poor prognosis (**Table 1**). Besides, multivariate analyses showed that *OIP5* overexpression (P = 0.037), age (*P* < 0.001), grade (*P* = 0.001) and stage (*P* = 0.046) were all independent predictors of an unfavorable prognosis (**Table 1**).

**Table 1:** Cox regression analysis for the overall survival rates of ccRCC patients.

Variable	Univariate analysis			Multivariate analysis		
	HR	95% CI	*P*	HR	95% CI	*P*
Age	1.033	1.019-1.047	<0.001	1.036	1.021-1.052	<0.001
Gender	0.931	0.675-1.284	0.663			
Grade	2.293	1.854-2.836	<0.001	1.496	1.176-1.904	0.001
Stage	1.889	1.649-2.164	<0.001	1.585	1.007-2.495	0.046
T	1.941	1.639-2.299	<0.001			
M	4.284	3.106-5.908	<0.001			
OIP5	2.622	1.832-3.751	<0.001	1.594	1.027-2.471	0.037

### The Landscape of Infiltrating Immune Cells in ccRCC and Normal Renal Tissues

**Figure 5:**
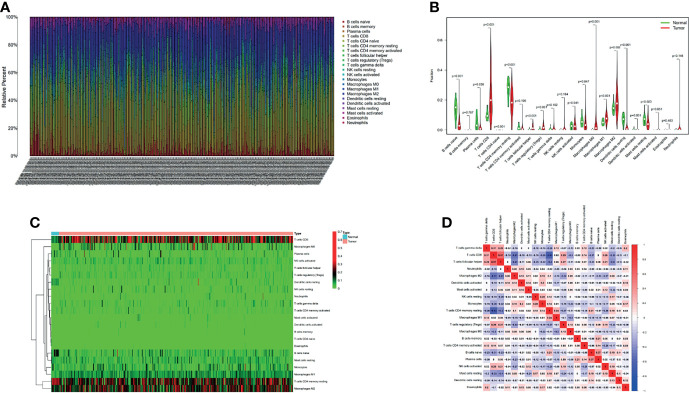
The proportion of 22 immune cell infiltrates was assessed using Cell Type Identification By Estimating Relative Subsets Of RNA Transcripts method in ccRCC cohort of TCGA **(A)**. Heatmaps and violin plots showed the differences in the immune cell distribution between malignant (red) and normal (blue) tissues in ccRCC cohorts **(B, C)**. Different correlation patterns among immune cell subsets in ccRCC cohorts **(D)**.

We first extracted and processed signature gene expression profiles using the CIBERSORT method to systematically describe the patterns of immune cells. After excluding samples with *P* ≥ 0.05, the landscape of immune cells infiltration in ccRCC and normal renal tissue were shown in **Figure 5A**. Compared to normal renal tissues, the proportions of T cells CD8, T cells follicular helper, T cells regulatory (Tregs), Macrophages M0 and Macrophages M1 were significantly overexpressed, while B cells naïve, Plasma cells, T cells CD4 naïve, T cells CD4 memory resting, Monocytes, Dendritic cells resting and Mast cells resting decreased in ccRCC (**Figure 5B**). **Figure 5C** showed different patterns of infiltrating immune cells in ccRCC compared with normal tissue. Further, different correlation patterns among immune cells were detected in ccRCC (**Figure 5D**).

### Relationship Between *OIP5* and Immunity

**Figure 6:**
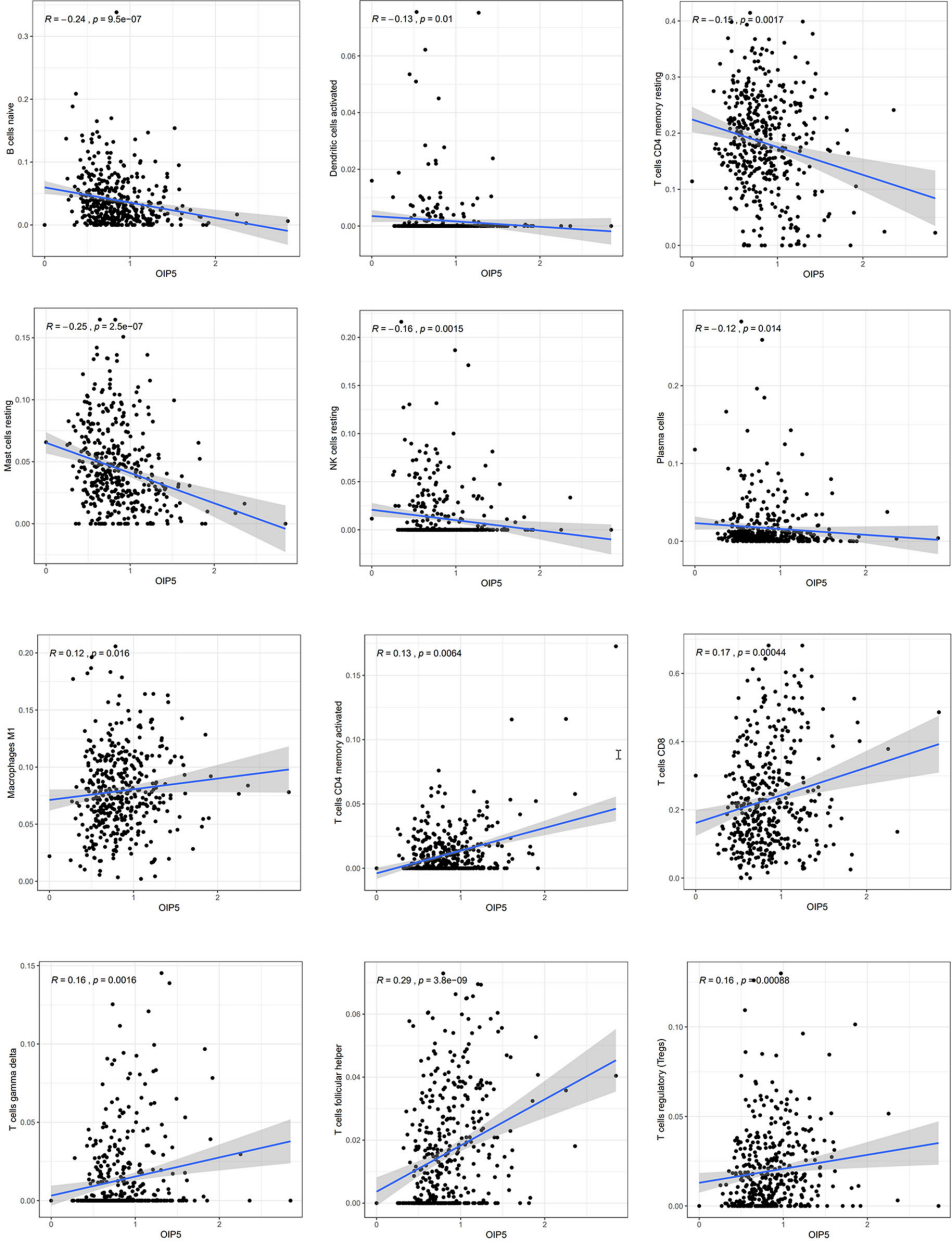
The relationships between *OIP5* expression levels and immune cell subsets in ccRCC.

**Figure 7:**
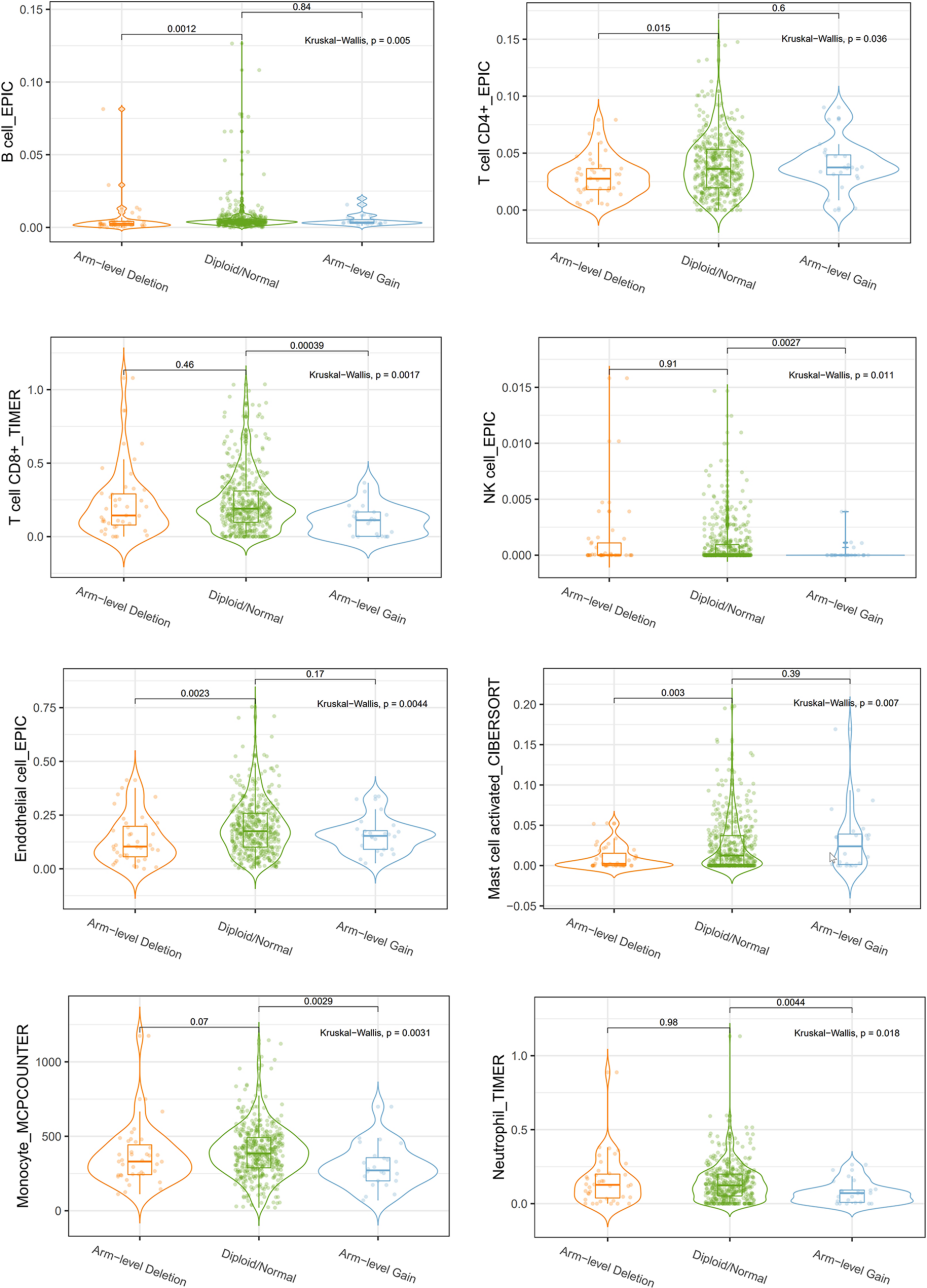
Correlations between *OIP5* gene copy numbers and immune cell infiltration levels.

We explored the Relationship between *OIP5* and the immune system. We found that the expression level of *OIP5* was positively correlated with T cells CD8 (*P* < 0.001), T cells CD4 memory activated (*P* = 0.006), T cells follicular helper (*P* < 0.001), T cells regulatory (Tregs) (*P* < 0.001), T cells gamma delta (*P* = 0.001) and Macrophages M1 (*P* = 0.016). While, the expression level of *OIP5* was negatively correlated with B cells naïve (*P* < 0.001), Plasma cells (*P* = 0.014), T cells CD4 memory resting (*P* = 0.001), NK cells resting (*P* = 0.001), Dendritic cells activated (*P* = 0.01) and Mast cells resting (*P* < 0.001) (**Figure 6**). At the same time, we found that the levels of several immune cell infiltration appeared to correlate with altered copy number of *OIP5* gene, including B cell, CD4^+^T cell, CD8^+^T cell, NK cell, T cell regulatory (Tregs), Neutrophil, Monocyte, Mast cell activated and Endothelial cell in ccRCC (**Figure 7**).

**Figure 8:**
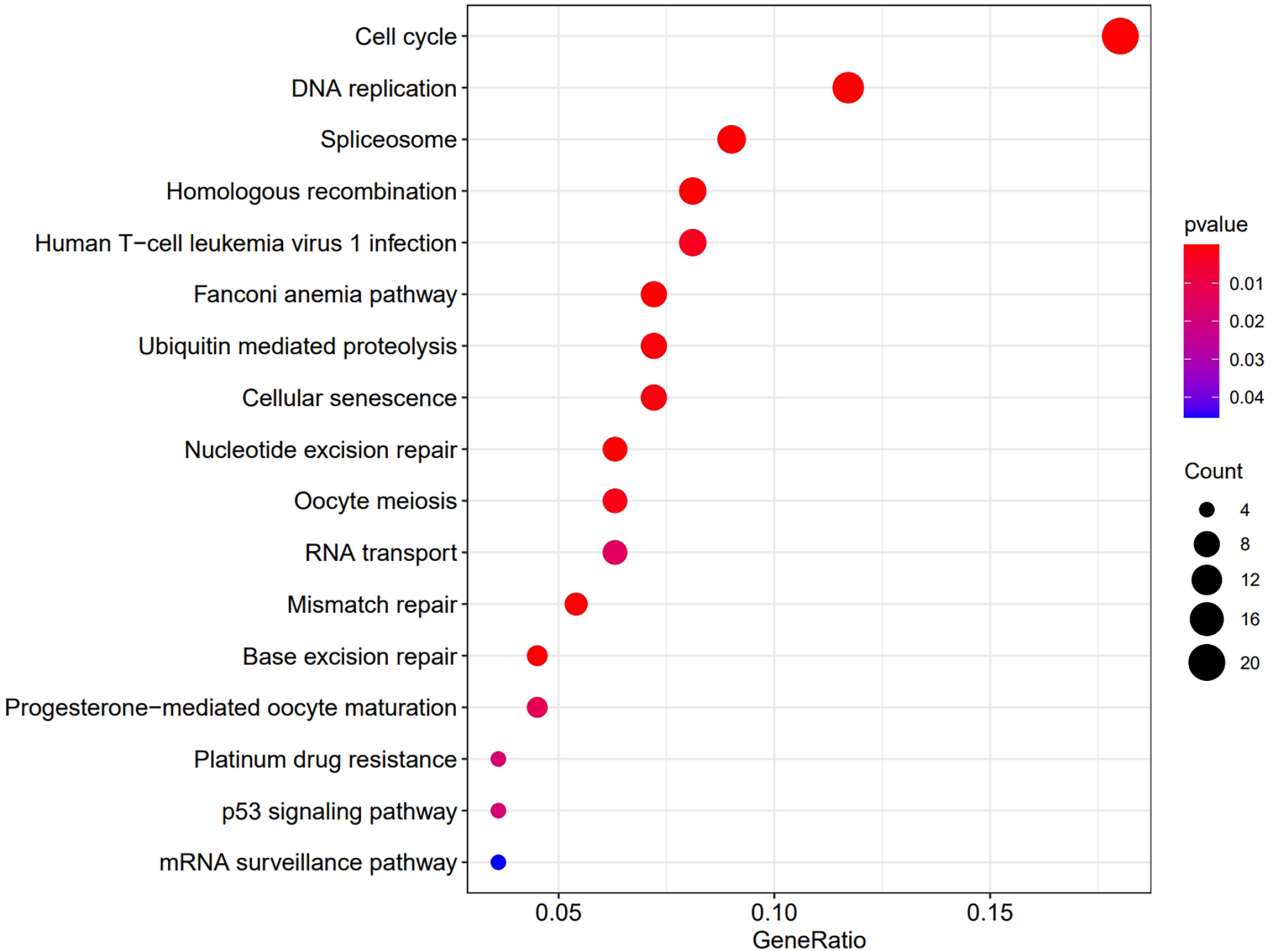
KEGG analysis shows the pathways which *OIP5* may regulate.

**Figure 9:**
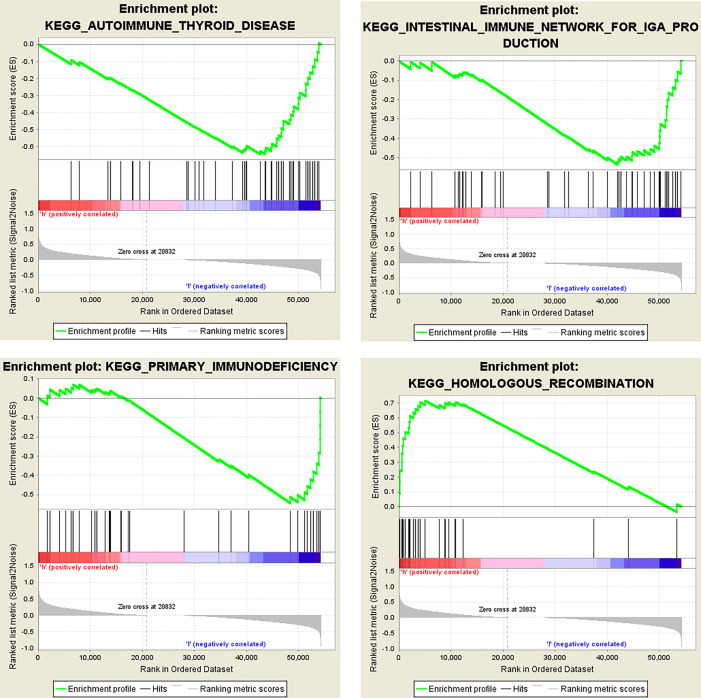
Dissection of *OIP5*-associated immune signaling pathways by Gene Set Enrichment Analysis of 32 ccRCC cell lines from the Cancer Cell Line Encyclopedia database.

Then we analyzed the RNAseq data of 32 ccRCC cell lines downloaded from the CCLE website. The KEGG results suggested that *OIP5* may regulate immunity through Homologous recombination, Human T−cell leukemia virus 1 infection, Fanconi anemia pathway and Nucleotide excision repair signaling pathway (**Figure 8**). Further, GSEA analysis showed that *OIP5* was associated with several immune-related signaling pathways, including the homologous recombination signaling pathway (NES = 1.75, *P* =0.004), autoimmune thyroid disease signaling pathway (NES = -1.68, *P* = 0.004), primary immunodeficiency signaling pathway (NES = -1.58, *P* = 0.021), and intestinal immune network for IgA production (NES = -1.54, *P* = 0.03) (**Figure 9**).

**Figure 10:**
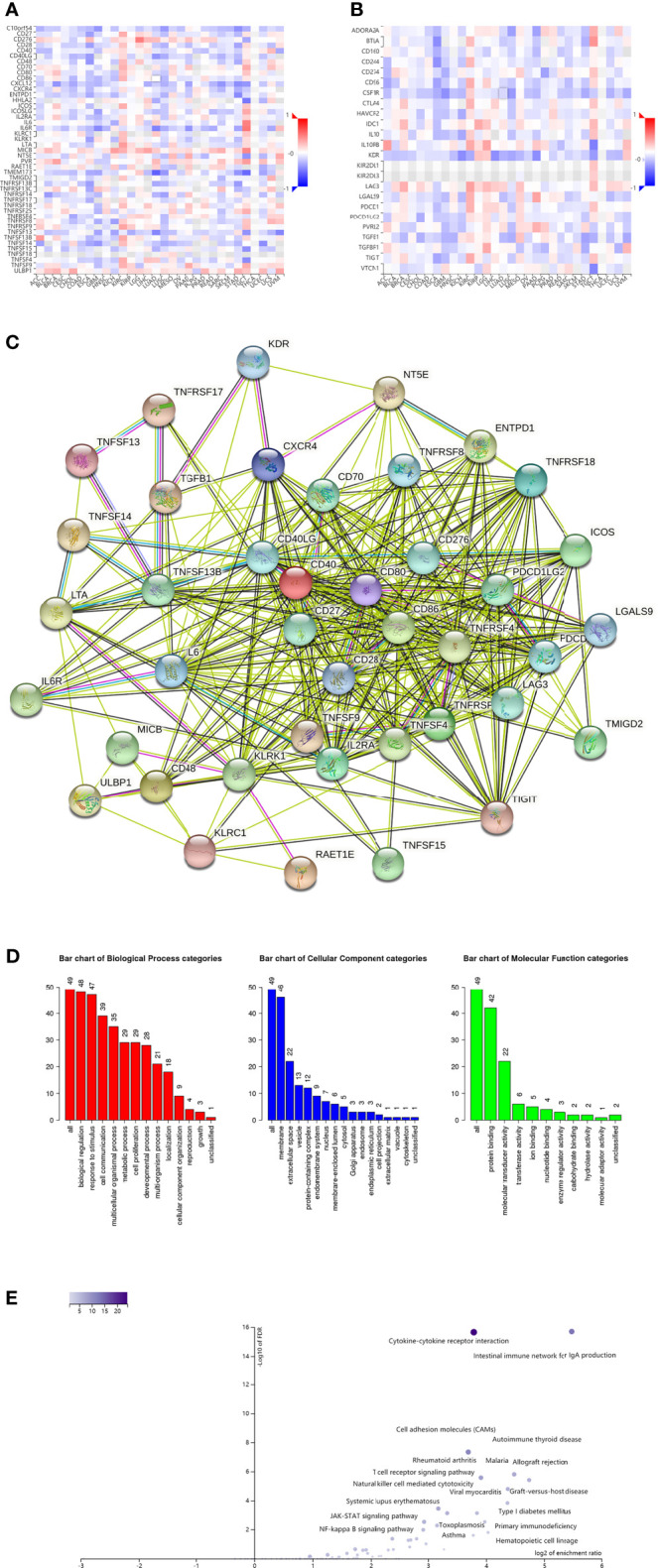
Identification and analysis of immunomodulators associated with the OIP5 gene. **(A)** The heatmap of relationship between the immunostimulators and the OIP5 gene in ccRCC. **(B)** The heatmap of relationship between the immunoinhibitors and the OIP5 gene in ccRCC. **(C)** Protein–protein network of 49 OIP5-associated immunomodulators in ccRCC, plotted by the STRING online tool. **(D)** Gene Ontology annotation of 49 OIP5-associated immunomodulators in ccRCC. **(E)** Kyoto Encyclopedia of Genes and Genomes pathway analysis of 49 OIP5-associated immunomodulators in ccRCC.

We further investigated the signaling pathways that *OIP5* may be involved in regulating the immune response to ccRCC. We identified 34 immunostimulators (CD27, CD28, CD40, CD40LG, CD48, CD70, CD80, CD86, CD276, CXCR4, ENTPD1, ICOS, IL2RA, IL6, IL6R, KLRC1, KLRK1, LTA, MICB, NT5E, RAET1E, TMIGD2, TNFRSF4, TNFRSF9, TNFRSF17, TNFRSF18, TNFSF4, TNFSF9, TNFSF13, TNFSF13B, TNFSF14, TNFSF15, ULBP1 and TNFRSF8) (**Figure 10A**) and 15 immunoinhibitors (BTLA, CD96, CD244, CSF1R, CTLA4, HAVCR2, IL10, IL10RB, KDR, LAG3, LGALS9, PDCD1, PDCD1LG2, TGFB1 and TIGIT) (**Figure 10B**) significantly associated with *OIP5* in ccRCC. We also screened the protein-protein interaction (PPI) network in the String database using these immunomodulators (**Figure 10C**). Next, we investigated the enriched GO terms and KEGG pathways using these immunomodulators (**Figures 10D, E**). The results of KEGG pathways showed that T cell receptor signaling pathway, primary immunodeficiency, Natural killer cell mediated cytotoxicity, NF-kappa B signaling pathway, JAK-STAT signaling pathway and Cytokine-cytokine receptor interaction were related to *OIP5*-mediated immune events (**Figure 10E**).

### The Effect of *OIP5*-Related Immunomodulators on Prognosis of ccRCC

**Figure 11:**
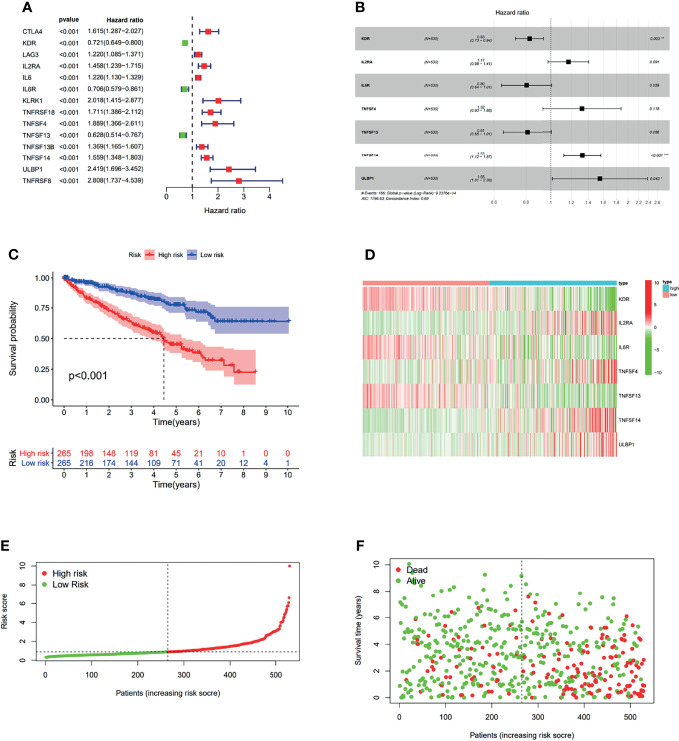
The development of prognostic gene signatures based on 49 *OIP5*-associated immunomodulators. The hazard ratios of genes integrated into the prognostic signatures are shown in the forest plots for ccRCC **(A, B)**. Kaplan–Meier curves for ccRCC regarding the risk scores **(C)**. Distribution of risk scores, along with survival statuses, and gene expression profiles for ccRCC **(D–F)**. *P < 0.05, **P < 0.005, ***P < 0.001.

In order to investigate the prognostic value of *OIP5*-related immunomodulators in ccRCC, the univariate Cox regression analysis was performed on these genes. We identified 14 genes that were significantly associated with the prognosis of ccRCC patients (**Figure 11A**). Then, multivariate stepwise Cox regression analysis was performed for these variables. This method produced an optimal 7-gene prognostic signature in ccRCC (**Figure 11B**). The risk scores were obtained by adding up the product of expression value and coefficient of each gene. The Kaplan-Meier survival curve demonstrated that patients with high-risk scores had significantly shorter survival than those with low-risk scores (log-rank test, *P* < 0.001) (**Figure 11C**). We also investigated the distributions of signature gene expression profiles, risk scores and survival statuses for ccRCC (**Figures 11D–F**).

## Discussion

In the United States, there are about 64,000 new cases and nearly 14,000 deaths due to renal cell cancer each year (1). Therefore, there is an urgent need to find an effective diagnosis and treatment for renal cancer. In the past decade, tumor microenvironment (TME) has rapidly attracted the attention of the oncology community, and is considered to be a key factor affecting the development, treatment resistance and prognosis of tumors (21–23). Previous studies have demonstrated that tumor-infiltrating immune cells (TICS) in TME can be used as a valid indicator of prognosis and therapeutic efficacy (24). In this research, we found that *OIP5* was closely related to the immunity of ccRCC. The expression of *OIP5* gene was related to the level of immune cell infiltration and immunomodulators. Using stepwise Cox regression model, we successfully obtained multi-gene risk prediction signals from *OIP5*-related immunomodulators.

Our results showed that *OIP5* was highly expressed in ccRCC and high expression of *OIP5* gene was correlated significantly with the patient’s age, the tumor histological grade, T classification, N classification, M classification and clinical stage, which was consistent with our previous findings (13). The results further confirmed the role of the *OIP5* as an oncogene in ccRCC. However, the specific mechanism of *OIP5* in ccRCC and the relationship between *OIP5* and immunity are still unclear. We evaluated the composition of intratumoral immune subsets in individual patients using CIBERSORT analysis, because some immunotherapies have been developed to modulate these cells. Our results showed that the composition of 22 immune subsets in the tumor microenvironment of ccRCC were significantly altered compared to normal kidney tissue. These results suggested that the pattern of intratumoral immune cell infiltration is related to the prognosis of ccRCC.

Importantly, we discovered that *OIP5*, a putative oncogene, was associated with immune cell infiltration in ccRCC. To our knowledge, this research first demonstrated an association between *OIP5* and ccRCC immunity. We found that *OIP5* gene copy number was correlated with infiltration levels of B cell, CD4^+^T cell, CD8^+^T cell, NK cell, T cell regulatory (Tregs), Neutrophil, Monocyte, Mast cell activated and Endothelial cell in ccRCC.

A deeper analysis of the complexity of TME may discover the advanced biomarkers that can help identify patient populations which respond to current immune checkpoint therapy and help identify new adjuvant therapy targets. The combination with immunotherapy drugs will be an important supplement to reduce the toxic side effects and improve the treatment effect of advanced ccRCC (25). The relationship between tumor cells and the immune system is determined by a complex network of cell–cell interactions. In addition, infiltrating stromal cells and immune cells are the main components of normal cells in tumor tissue. So, the specific immune cell composition of the tumor was closely related to patient’s prognosis. Our research demonstrated that *OIP5* could change the components of immune cells of ccRCC.

Further, a KEGG pathway analysis of *OIP5*-associated immunomodulators showed that the primary immunodeficiency pathway, the JAK-STAT signaling pathway, the T cell receptor signaling pathway and the NF-kappa B signaling pathway might be involved in *OIP5*-mediated immune response. Previous research revealed that *OIP5* could promote metastasis of nasopharyngeal carcinoma cells by promoting EMT *via* modulation of JAK2/STAT3 signal (26). Although several mechanisms have been identified that prevent the generation of anti-tumor immune responses, the one that has attracted the most attention is the expression of key receptors on the surface of T cells, which prevent full activation of T cells (27–31). In conclusion, it is speculated that *OIP5* inhibitors may also enhance tumor immunity from a biological perspective, thus contributing to the anti-tumor efficacy of immune checkpoint blockers.

Consistently, we constructed immune gene signatures for ccRCC with *OIP5*-associated immunomodulators. The risk scores derived from the gene signatures were dramatically associated with the survival of ccRCC patients. Our research showed that the risk scores derived from *OIP5*-associated immunomodulators could distinguish risk groups identified by a differential expression of a set of signature genes. The results of our study may improve the development of well-verified signatures for the prognosis of ccRCC.

Despite some merits of our study, there still exists several shortcomings. First, the analysis results from TCGA have not been detected in GEO database. Second, all the analyses were conducted with public datasets. Thus, if possible, further experiments should be conducted *in vivo* and *in vitro* to certificate these results.

In general, this study revealed an important relationship between *OIP5* gene and tumor immune microenvironments. The prognostic signatures derived from *OIP5*-associated immunomodulators were independently predictors of overall survival in ccRCC. More studies are needed to verify the oncogenic role and potential mechanism of *OIP5* in ccRCC, and more cohorts are needed to improve the predictive accuracy then help the personalized management of ccRCC patients.

## Data Availability Statement

The datasets presented in this study can be found in online repositories. The names of the repository/repositories and accession number(s) can be found in the article/supplementary material.

## Author Contributions

All authors listed have made a substantial, direct, and intellectual contribution to the work, and approved it for publication.

## Funding

Supported by Science and Technology Plan Project of Zhongshan City 2019B1064, Science and Technology Plan Project of Zhongshan City 2018B1031 and Major Science and Technology Plan project of Zhongshan City 2016B1003.

## Conflict of Interest

The authors declare that the research was conducted in the absence of any commercial or financial relationships that could be construed as a potential conflict of interest.

## Publisher’s Note

All claims expressed in this article are solely those of the authors and do not necessarily represent those of their affiliated organizations, or those of the publisher, the editors and the reviewers. Any product that may be evaluated in this article, or claim that may be made by its manufacturer, is not guaranteed or endorsed by the publisher.
